# Treatment of calcinosis cutis by extracorporeal shock‐wave lithotripsy—A patient experience

**DOI:** 10.1002/ski2.397

**Published:** 2024-05-29

**Authors:** Cristina Grechin, Nicola Kearney, Muireann Roche

**Affiliations:** ^1^ Dermatology Department Beaumont Hospital Dublin Ireland

## 1 PATIENT VIEWPOINT

I have had scleroderma for many years and later on I ended up being diagnosed with calcinosis cutis (CC). To be honest, I didn't feel awful about it because I have always been in hospitals. Since I was a child, I have been in and out of hospital all those years. I kind of take things in my stride. It was just another thing that I had to deal with it.

For over 10 years, I have been troubled by calcinosis in my arms and fingertips. My arms gave me a lot of trouble a few years ago. One of the main problems was trying to fight infection when calcinosis caused wounds. When it first got infected and it opened up, that one carried on for 1 year, and I had to dress it every day, going back and forth to the clinic. After multiple failed treatments, my doctor decided to offer me lithotripsy. I was happy to start it as is non‐invasive and what I was going to lose if I would try it. My arms have completely settled down and I have had no wounds on them for about 3 years. Fortunately, lithotripsy keeps from getting inflamed or infected. That's a good thing. It is an improvement‐probably it doesn't look visually an improvement but generally it does keep the calcinosis down. If I didn't have the lithotripsy it may be bigger and it be back where it was initially. It's a slow process to see the results, but I would certainly recommend it for other people in my situation. It takes about 15 min, is relatively pain free and requires no needles, which I greatly appreciate.

## 2 CLINICIAN VIEWPOINT

CC is a rare, debilitating complication of connective tissue disease and chronic venous insufficiency.^1^ This condition is characterised by deposition of insoluble calcium salts in the skin and subcutaneous tissues, often associated with severe pain, reduced mobility, chronic infections and significantly decreased quality of life.^2^

Extracorporeal shockwave lithotripsy (ESWL) is a minimally invasive procedure with a low risk of adverse effects that has been described in the literature as an effective treatment option for conditions including urolithiasis, pancreato‐lithiasis, and calcified tendonitis.^1^ More recently, ESWL has been described as an effective treatment modality for recalcitrant CC, being effective against small, ulcerated, and radiopaque CC.^1^, ^3^ ESWL also conveys an analgesic effect that is thought to make subsequent surgical excision of CC fragments easier.^1^, ^3^

This case illustrates a case of recalcitrant dystrophic CC (previously failed prolonged oral tetracyclines, rifampicin in association with clindamycin, diltiazem, bisphosphonates etc.) that has been successfully treated with five sessions of ESWL (1200 shocks per session) over a 5 year period, inducing and maintaining remission. The patient tolerated the procedures well, without developing any complications. This novel therapeutic approach led to an improvement in this patient's quality of life due to a reduced rate of recurrent infections and hospital admissions. As ESWL is a novel practice in dermatology, there is an absence of literature commenting on complications of ESWL in CC. However, it has been shown in other specialties to be a safe and non‐invasive treatment.^4^, ^5^

Furthermore, the efficacy of this treatment in terms of pain reduction and epithelialization, easy application, safety, and tolerability, should encourage other clinicians and patients to consider it as a minimally invasive treatment option for CC. This case highlights the positive impact that ESWL, a novel therapeutic approach for the treatment of CC, has had on this patient's quality of life. It also conveys the tolerability of this minimally invasive treatment option from a patient's perspective.

## CONFLICT OF INTEREST STATEMENT

None to declare.

## AUTHOR CONTRIBUTIONS

**Cristina Grechin**: Conceptualization (equal); project administration (equal); visualization (equal); writing – original draft (equal). **Nicola Kearney**: Conceptualization (equal); writing – review & editing (equal). **Muireann Roche**: Conceptualization (equal); supervision (equal); writing – review & editing (equal).

## ETHICS STATEMENT

Not applicable.

## PATIENT CONSENT

Fully informed written patient consent for publication was obtained.

## Data Availability

No new data were generated or analysed in support of this research.
